# 10 Years of Severe Acute Respiratory Infections Network (SARInet plus): Accomplishments and Way Forward

**DOI:** 10.1093/infdis/jiaf039

**Published:** 2025-03-10

**Authors:** Marc Rondy, Eduardo Azziz-Baumgartner, Rebecca Kondor, Rakhee S Palekar, Wenqing Zhang, Andrea S Vicari

**Affiliations:** Infectious Hazards Management Unit, Health Emergencies Department, Pan American Health Organization, Washington, DC; Influenza Division, National Center for Immunization and Respiratory Diseases, US Centers for Disease Control and Prevention, Atlanta, Georgia; Influenza Division, National Center for Immunization and Respiratory Diseases, US Centers for Disease Control and Prevention, Atlanta, Georgia; Health and Society Innovation Center, The MITRE Corporation, McLean, Virginia; Global Influenza Programme, Epidemic and Pandemic Preparedness, WHO Emergency Programme, World Health Organization, Geneva, Switzerland; Infectious Hazards Management Unit, Health Emergencies Department, Pan American Health Organization, Washington, DC

**Keywords:** Severe Acute Respiratory Infections Network (SARInet plus), respiratory virus surveillance, pandemic preparedness, genomic surveillance, influenza vaccine effectiveness

Acute respiratory infections are a leading cause of illness and death worldwide [1]. Because of the health and economic impact, the prevention of and preparedness for their seasonal epidemic and pandemic occurrence are a priority. The pandemics due to influenza virus A(H1N1)pdm09 and SARS-CoV-2 have highlighted the importance of international public health networks in preventing, detecting, and responding to these emergencies [2, 3]. Such networks create a community for sharing information, best practices, and insights among countries. The Severe Acute Respiratory Infections Network (SARInet plus) is a prime example of such a collaboration and cooperation mechanism. It was established in 2014 to strengthen surveillance and response capacities for acute respiratory infections in the Americas. This regional network fosters collaboration among epidemiologists, virologists, clinicians, and other public health professionals across the 35 member states in the Americas, the Pan American Health Organization (PAHO), the US Centers for Disease Prevention and Control, and other international partners.

Since its creation 10 years ago, SARInet plus has achieved significant milestones. Laboratory capabilities across member states have substantially improved, with the recognition by the World Health Organization (WHO) of the most National Influenza Centers worldwide and a widespread introduction and quality use of molecular diagnostics and genomic sequencing across the region. As highlighted by Born and colleagues [4] in this theme issue, the SARInet plus laboratory network currently includes 31 National Influenza Centers and 13 national reference laboratories, which report virologic data on influenza and other respiratory virus detection weekly to the global platform FluNet. By leveraging this decade-long progress, countries were able to adopt genomic surveillance capacities at the inception of the COVID-19 pandemic, which have now led to the establishment of the PAHOGen network, a genomic surveillance platform for the Americas aiming to enhance genomic surveillance and sequencing capabilities within the countries and across pathogens [5]. Sharing of sequencing data from circulating viruses is necessary to inform influenza vaccine composition. Southern Hemisphere data from countries in our region are critical for the WHO Global Influenza Surveillance and Response System to analyze patterns and identify viruses that are considered in the seasonal formulation of influenza vaccines.

Surveillance data generated within SARInet plus are also used to generate policy-relevant findings such as the annual estimates of seasonal influenza vaccine effectiveness. Fifteen Latin American and Caribbean countries are part of the Network for the Evaluation of Vaccine Effectiveness in Latin America and the Caribbean–influenza (REVELAC-i), an initiative established in 2013 that provides evidence to guide immunization decision making [6]. The aim of REVELAC-i is for countries to share knowledge on influenza vaccine effectiveness and generate evidence based on epidemiologic, virologic, and immunization data to support influenza prevention and control. The collaborative efforts between SARInet plus and REVELAC-i have bolstered the region's capacity to use evidence to address respiratory threats effectively and provide genomic surveillance and vaccine effectiveness data to inform the biannual influenza strain selection committee.

Effective pandemic preparedness is built during interepidemic phases. The strength of networks such as SARInet plus was particularly evident during the COVID-19 pandemic. The rapid integration of SARS-CoV-2 molecular testing first and surveillance later into the already-existing network facilitated a timely, coordinated response to the pandemic [2]. As highlighted by Rodriguez and colleagues [7], this response was reinforced through continuous education sessions, including webinars, workshops, and regional meetings channeled through SARInet plus. Following WHO recommendations, most countries in the Americas implemented comprehensive universal COVID-19 surveillance strategies. To support countries’ decision in finding more sustainable surveillance, Redondo-Bravo and colleagues report findings showing that integrating SARS-CoV-2 into existing sentinel systems for respiratory viruses is a viable approach for monitoring viral and epidemiologic characteristics of acute respiratory infections [8].

Another key strength of SARInet plus is the exchange and use of surveillance data to guide influenza vaccination programs in the region. The study by Descalzo and colleagues illustrates the importance of measuring the burden of influenza disease for decision makers [9]. For instance, their findings show that in 6 South American countries (with an estimated population of 307 million people), the yearly influenza-associated burden of disease ranged from 51 to 78 million cases of mild to moderate influenza, from 323 000 to 490 000 influenza-associated hospitalizations, and from 22 600 to 47 000 influenza-associated deaths during the 2015–2019 influenza seasons [9]. Metrics of burden of disease can help countries prepare their public health response and promote the implementation of effective prevention programs. These data can also be used to estimate the number of illnesses averted through vaccination programs, such as the analysis presented by Jara and colleagues [10]. Countries can further use these metrics to get an estimate of the health and economic impact of influenza vaccination and predict what vaccination campaigns would be the most effective.

Although these accomplishments have significantly strengthened respiratory virus surveillance in the region of the Americas, some challenges demand continued attention. One of the most pressing needs is a reliable capacity for early detection and rapid response to pandemic threats [7]. To improve these capacities, intersectoral initiatives and enhanced surveillance strategies at the human-animal-environmental interface are vital. Recent increases of avian influenza outbreaks in wild and farm animals and reports of human cases in the United States, Canada, Ecuador, and Chile underscore the need for a robust intersectoral collaboration and response [11]. At the request of regional thought leaders, PAHO and partners launched the Intersectoral Technical Commission for the Prevention and Control of Zoonotic Influenza in the Americas in March 2024 to foster intersectoral initiatives, facilitate knowledge exchange, coordinate regional strategies, promote operational research, implement informed actions, and guide countries in implementing concrete One Health approaches to tackle pandemic threats. Additionally, to strengthen our capacity to detect, assess, and respond early to a pandemic threat, PAHO has been working on guidance to implement unusual respiratory event surveillance. Sharing experiences in the implementation of these initiatives through SARInet plus will be an important focus in the coming years.

To be ready for the next pandemic, it is important for countries to count on robust pandemic preparedness and response plans. These plans must be comprehensive, updated regularly, and realistic based on existing national capacities and resources. As shown by Villalobos and colleagues [12], current national plans from countries in the Americas lack alignment with global guidance. Therefore, countries need to urgently revise their pandemic preparedness and response plans and incorporate lessons from past pandemics. Initiatives such as the WHO's Preparedness and Resilience for Emerging Threats provide targeted guidance, training, simulation exercises, and other resources to support countries in updating their national pandemic plans [13].

Moving forward, SARInet plus will continue to foster collaboration; regional harmonization in surveillance and laboratory protocols and practices; and mutual knowledge sharing through annual meetings, webinars, working group activities, and the distribution of reference documents and guidelines. As we confront the ongoing challenge of potential emerging pandemics, the continued strengthening and consolidation of SARInet plus will be crucial to protect the people of the Americas. As new vaccines against respiratory viruses are being introduced (eg, against respiratory syncytial virus), SARInet plus will provide an ideal platform to monitor the epidemiologic, virologic, and economic impact of these public health interventions. PAHO, as SARInet plus secretariat, will remain vigilant in eliciting the resources that this network needs to thrive. Nevertheless, the true strength and impact of SARInet plus lie in its members and international partners, and the active engagement and continuous involvement from national health authorities will be key to keeping the network relevant and effective.

## Notes

***Acknowledgments*.** This article appears as part of the supplement “Advancing Respiratory Virus Surveillance and Preparedness in the Americas: A Decade of SARInet's Impact,” sponsored by the PAHO, which reflects the work of the SARInet plus members. We thank all of them for their work and commitment over the past 10 years. These include colleagues from the PAHO regional offices—in particular, P. Born, M. Cerpa, M. J. Clari, P. Couto, M. Descalzo, J. Jara, J. Leite, J. Mendez-Rico, R. Ramirez, L. Redondo, A. Rodriguez, C. Serrano, A. Smith, A. Villalobos, I. Wellington—and country offices, the WHO, the US CDC, and the US Department of Health and Human Services, as well as representatives from the ministries of health of Antigua and Barbuda, Argentina, Bahamas, Barbados, Belize, Bolivia, Brazil, Canada, Chile, Colombia, Costa Rica, Cuba, Dominica, Dominican Republic, Ecuador, El Salvador, Grenada, Guatemala, Guyana, Haiti, Honduras, Jamaica, Mexico, Nicaragua, Panama, Paraguay, Peru, Saint Kitts and Nevis, Saint Lucia, Saint Vincent and the Grenadines, Suriname, Trinidad and Tobago, United States, Uruguay, and Venezuela. We also thank Olaya Astudillo for her editing work on all articles from this special issue.

***Disclaimer*.** The content of this report is solely the responsibility of the authors and does not necessarily represent the official views of the Pan American Health Organization (PAHO). The findings and conclusions in this report are those of the authors and do not necessarily represent the official position of the Centers for Disease Control and Prevention. The findings and conclusions in this report are those of the authors and do not necessarily represent the official position of the MITRE Corporation.

***Financial support*.** This work was partially supported by a grant from the Centers for Disease Control and Prevention (CDC) through the Pan American Health Organization (PAHO) under Cooperative Agreement Number NU51IP000940, as well as a grant from the World Health Organization (WHO) under the Pandemic Influenza Preparedness Framework.

***Supplement sponsorship.*** This article appears as part of the supplement “Advancing Respiratory Virus Surveillance and Preparedness in the Americas: A Decade of SARInet's Impact,” sponsored by the Pan American Health Organization.

***Potential conflicts of interest***. All authors: No reported conflicts. All authors have submitted the ICMJE Form for Disclosure of Potential Conflicts of Interest.
